# Carers for older people with co-morbid cognitive impairment in general hospital: characteristics and psychological well-being

**DOI:** 10.1002/gps.3871

**Published:** 2012-08-09

**Authors:** Lucy E Bradshaw, Sarah E Goldberg, Justine M Schneider, Rowan H Harwood

**Affiliations:** 1 Division of Epidemiology and Public Health, University of NottinghamNottingham, UK; 2 Division of Rehabilitation and Ageing, University of NottinghamNottingham, UK; 3 School of Sociology & Social Policy, University of NottinghamNottingham, UK; 4 Health Care of Older People, Nottingham University Hospitals NHS Trust, Queen's Medical CentreNottingham, UK

**Keywords:** carers, older person, mental health, general hospital

## Abstract

**Objective:**

This analysis sought to describe the characteristics and well-being of carers of older people with mental health problems admitted to a general hospital.

**Methods:**

General medical and trauma orthopaedic patients aged 70 years or older admitted to an acute general teaching hospital were screened for mental health problems. Those screened positive, together with a carer, were invited to undergo further assessment with a battery of health status measurements. Carers were interviewed to ascertain strain (caregiver strain index (CSI)), psychological distress (12-item General Health Questionnaire) and quality of life (EQ-5D).

**Results:**

We recruited 250 patients to the study, of whom 180 were cognitively impaired and had carers willing to take part. After 6 months, 57 patients (32%) had died, and we followed up 100 carers. Carers' own health, in terms of mobility, usual activities, and anxiety, was poor in a third of cases. At the time of admission, high carer strain was common (42% with CSI ≥ 7), particularly among co-resident carers (55%). High levels of behavioural and psychiatric symptoms at baseline were associated with more carer strain and distress. At follow-up, carer strain and distress had reduced only slightly, with no difference in outcomes for carers of patients who moved from the community to a care home.

**Conclusion:**

Hospital staff should be alert to sources of carer strain and offer carers practical advice and emotional support. Interventions are required to prevent and manage behavioural and psychiatric symptoms at the time of acute physical illness or to alleviate their effects on carers. Copyright © 2012 John Wiley & Sons, Ltd.

## Introduction

Informal carers are family or friends who provide regular support and assistance to another adult who is ill, disabled or frail. Their role is recognised as making an important contribution to health and social care. Without them, the demand for professional care would be far greater. In the UK, government policy aims to improve support for carers to prevent or delay admissions to hospital and care homes (Department of Health, 2010b).

A growing number of older people with cognitive impairment living in the community rely on informal carers. When these patients are admitted to hospital for physical health problems, carers may not be much involved or assessed (Alzheimer's Society, 2009; Bridges *et al*., 2010) although carers' well-being is proposed as an indicator of quality of care (Department of Health, 2010a). Care home placement is more frequent, and length of hospital stay is longer for patients with co-morbid mental health problems than for patients without (Holmes and House, 2000; Sampson *et al*., 2009), and carers' subjective experience or burden may contribute to these outcomes. Carers of frail older people are prone to psychological distress (Buck *et al*., 1997; Waite *et al*., 2004; Idstad *et al*., 2010). Spouse carers of people with dementia have a four-fold risk of depression compared with spouses of people without dementia (Joling *et al*., 2010). Carers' psychological distress correlates with their perceived need for support from services (Stirling *et al*., 2010) and is amenable to tailored interventions (Hoskins *et al*., 2005).

This analysis sought to describe the carers of older people with cognitive impairment (delirium and dementia) who had unplanned admissions to a general hospital. We measured carer well-being, which we operationalised as carer strain, psychological distress and quality of life, and how it changed over 6 months following the hospital admission. Our hypothesis was that patient characteristics and carer living arrangements determine carer well-being.

## Methods

### Study population

Patients aged 70 years or older with an unplanned admission lasting two or more days to one of 12 acute general hospital wards (two trauma orthopaedic, three acute geriatric medical and seven general medical) from two sites of an 1800-bed teaching hospital were screened for mental health problems by using brief tests of cognition (Hodkinson, 1972), depression (Almeida and Almeida, 1999), anxiety (Spitzer *et al*., 1994), alcohol misuse (Ewing, 1984) and a question asking if there was any other reason to believe a mental health diagnosis might be present. Patients screening positive were invited to take part in the study. Participants with mental capacity gave written informed consent. Those lacking mental capacity were recruited subject to agreement from a family member or carer.

Carers were separately invited to participate in this study, subject to informed consent. A carer was defined as ‘someone who has regular contact with the patient participant for at least an hour a week’. If there was more than one person who cared for the patient, either the family decided the most appropriate carer or the most available carer was approached.

The study was approved by the Bradford Research Ethics Committee.

### Measures

Baseline information on participants was collected by interview with the participant, carer informants and observation, and comprised the following: demographic details; medications taken at admission, co-morbidity (Charlson co-morbidity index; Charlson *et al*., 1987); severity of acute illness (Modified Early Warning Score; Subbe *et al*., 2001); presenting geriatric syndromes; cognitive function (Mini-mental state examination (MMSE); Folstein *et al*., 1975); delirium diagnosis and severity (Delirium Rating Scale–Revised–98; Trzepacz *et al*., 2001); depression (Cornell Scale for Depression in Dementia; Alexopoulos *et al*., 1988a; Alexopoulos *et al*., 1988b); behavioural and psychiatric symptoms (Neuropsychiatric Inventory (NPI); Cummings, 1997); activities of daily living (ADL) at admission and prior to the acute illness (Barthel Index; Mahoney and Barthel, 1965); nutritional status (short form Mini-Nutritional Assessment; Kaiser *et al*., 2009); and health-related quality of life (EuroQol EQ-5D; Brazier *et al*., 1996).

Carers were asked to complete a questionnaire, with help if required, comprising demographic and care giving details. This included the caregiver strain index (CSI) (Robinson, 1983), the 12-item General Health Questionnaire (GHQ12) to measure carer psychological distress (Goldberg and Williams, 1988) and the EuroQol EQ-5D to measure carer quality of life. Further details of these scales are given in Box 1.

**Box 1: Description of scales used in study**

For carers

- CSI—index comprising yes/no responses to 13 common stressors for people involved in informal caregiving including: adjustments to family, work and personal life; being upset by changes in behaviour or compared with how the person they are caring for used to be; disturbed sleep; and feeling overwhelmed. Questions were asked about the few weeks before the admission. The total strain score is calculated as the number of yes responses (range 0 to 13). Higher scores indicate greater strain, and a score of 7 or more indicates high strain.
- GHQ12—a tool developed to detect minor short-term psychiatric disorders and now commonly used in research studies to assess mental well-being/distress. The 12 questions are asked in relation to what is usual for the person completing the questionnaire. There are four possible responses for each question corresponding to better than usual, same as usual, worse or much worse than usual (wording depending on question). In this study, these four options were scored 0 to 3, respectively (Likert scoring). The total GHQ-12 score ranged from 0 to 36 with higher scores indicating increased psychological distress.

For participants

- NPI—tool to assess 12 common behavioural and psychiatric problems in patients with dementia in the 4 weeks prior to assessment, using information provided by a caregiver. Symptoms assessed are delusions, hallucinations, agitation, depression, anxiety, elation, apathy, disinhibition, motor behaviour disturbances, difficulty sleeping and appetite problems. A screening question first identifies if patients have a particular symptom, and if so, the frequency (occasionally, often, frequent, very frequent; coded 1 to 4) and severity (mild, moderate, marked; coded 1 to 3) are rated. A total summary score is calculated as the sum of frequency × severity scores for the 12 symptoms and ranges from 0 to 144, where higher scores indicate greater presence of behavioural and psychiatric problems.

### Follow-up

Participants and carers were followed up 180 days after recruitment. Bereaved family carers were not followed up. Carers were again asked to complete a questionnaire to assess their strain (CSI) and psychological distress (GHQ12) as well as to provide information on the participant's behavioural and psychiatric symptoms (NPI) and ADL. Information on care home placements for the patient participants during the follow-up period were ascertained from the carer informant, care home or the participants' general practitioner.

### Analysis

Participant and carer characteristics at recruitment were summarised overall and according to carer residence in relation to the participants: living apart from the carer, with the carer or in a care home (residential or nursing). Chi-squared tests and Kruskal–Wallis tests were used to test for differences in carer and patient characteristics at recruitment between these groups.

The association between carer strain (CSI) and psychological distress (GHQ12) at baseline and the participant characteristics (age, gender, admission ward, co-morbidity, cognitive impairment, delirium, ADL, incontinence and behavioural and psychiatric symptoms (using frequency and severity categories of individual symptoms, categories with low frequencies were grouped together, and the total summary score)) were explored. Negative binomial regression was used for the total number of ‘yes’ responses to the CSI. The distribution of the total GHQ12 score was right skewed: linear regression on the log transformed score was therefore used for the analysis. Associations were examined both univariately and including carer age, sex and residence in the models: the magnitude and direction of effect were similar for these, so the adjusted regression coefficients are presented. Some carers did not complete all items on the CSI and GHQ12 scales (18 for CSI, 10 with one item not completed and 8 for GHQ12, 3 with one item not completed). Analyses using simple imputation methods for these missing items gave similar results: results from the cases with complete data are therefore presented. Regression coefficients from the models were exponentiated to show the proportional (factor) change in scores for each category relative to the reference category shown by 1.

Changes in strain, psychological distress and quality of life for carers of surviving participants were summarised overall, according to residence in relation to the participant at recruitment and according to whether participants living in the community at admission moved to new permanent care homes.

Analyses were conducted in Stata version 11 (Statacorp, College Station, TX).

## Results

### Screening and recruitment

Between April and November 2009, there were 3680 unplanned admissions lasting more than 2 days of people older than 70 years to the study wards. One thousand and four patients were screened (Appendix 1), with 643 (64%) screening positive for a mental health problem. We identified no differences between those who were screened and those who were not (Goldberg *et al*., 2012). Of those screening positive, 61 (9%) had no identifiable carer, and 108 (17%) were excluded because they did not have capacity to give informed consent and we were unable to contact or meet with their documented carer prior to the patient's discharge. Two hundred and fifty patients were recruited; carers of these people were contacted, and 201 consented to participate in the carer study. Some patients with depression alone were relatively cognitively unimpaired (MMSE ≥ 24); carers of these patients were excluded from this analysis (*n* = 21). Of the 180 dyads included in our final analysis sample, 40% of the patients lived apart from their designated carer (*n* = 71), 32% lived together (*n* = 58) and 28% of the patients lived in a care home (*n* = 51).

### Patient characteristics

At admission, patients were very dependent on ADL. Half required major help transferring, half needed help feeding, half were incontinent and 64% had a Barthel Index ≤ 10/20 (≤50/100). More than half had delirium, and behavioural and psychiatric symptom (NPI) scores were high (Table 1). Some patient behaviours were more common than others: 60% showed apathy, 37% each showed agitation or aberrant motor behaviour, half had some evidence of delusions and a third had hallucinations (Table 3).

**Table 1:** Characteristics of patients at recruitment, by carer residence

	Community patients			
			
	Care home patients (*n* = 51)	Total (*n* = 180)	Carer lives elsewhere to patient (*n* = 71)	Carer lives with patient (*n* = 58)
Patients				
Male^a^	20% (14)	62% (36)	20% (10)	33% (60)
Age^a^	87 (83–92)	81 (78–86)	88 (84–92)	86 (81–90)
Charlson score^a^	2 (1–3)	3 (2–5)	2 (1–3)	2 (1–4)
Barthel ADL prior to current illness^a^	17 (13–18)	14 (9–18)	11 (8–16)	15 (10–18)
Barthel ADL at admission^a^	10 (7–13)	8 (5–13)	4 (1–7)	8 (4–13)
Incontinent of bladder at admission^b^	40% (28)	55% (32)	73% (37)	54% (97)
Incontinent of faeces at admission^b^	17% (12)	29% (17)	57% (29)	32% (58)
Cognitive function (MMSE) b	15.5 (11–20)	14 (10–19)	4 (0–10)	13 (6–18)
Delirium^b^,^c^	49% (34)	45% (26)	84% (43)	57% (103)
Total NPI score	24 (12–38)	25.5 (16–40)	32 (17–50)	26 (14–41)

Care delivered by carers				
Hours of physical care per day^a^				
Median (IQR)	2 (0.3–4)	18 (4–24)	0 (0–0)	2 (0–8)
Missing	12	7	3	22
Providing 12+ hours of physical care per day^a^	3% (2)	52% (30)	4% (2)	19% (34)
Hours of supervision per day^a^				
Median (IQR)	2 (0.3–4)	24 (14–24)	0 (0–0.6)	2 (0–22)
Missing	14	7	4	25
Providing 12+ hours of supervision per day^a^	4% (3)	69% (40)	4% (2)	25% (45)
No other unpaid carers^a^	55% (39)	79% (46)	88% (45)	72% (130)

*Note*: Median (IQR) are presented for continuous/ordinal variables and percentage (*n*) for categorical variables.

a Significant difference (*p* < 0.05) between three carer groups and two groups caring for community dwelling participants.

b Significant difference (*p* < 0.05) between three carer groups.

c Delirium Rating Scale score greater than 17.75.

**Table 3:** Association between carer strain and distress scores (GHQ12) at baseline and patient characteristics with adjustment for carer characteristics

		Carer strain score		GHQ12 score (distress)	
			
			Adjusted^b^ proportional change (95% CI)		Adjusted^b^ proportional change (95% CI)
					
Variable	Freq^a^	Median (IQR)	*n* = 159–161	Median (IQR)	*n* = 169–172
Cognitive impairment			*p* = 0.37		*p* = 0.49
Mild CI (21–24)	27 (15%)	6 (3–8)	1.00	12 (9–19)	1.00
Moderate CI (10–20)	85 (47%)	6 (3–9)	1.26 (0.91, 1.75)	14 (9–19)	1.08 (0.88, 1.31)
Severe CI (MMSE < 10)	67 (37%)	4 (2–9)	1.27 (0.87, 1.84)	13 (10–18)	1.14 (0.91, 1.43)

Incontinent of faeces at admission			*p* = 0.41		*p* = 0.03
Continent	89 (50%)	5 (2–8)	1.00	12 (9–17)	1.00
Occasional accident	32 (18%)	6 (3–10)	1.15 (0.85, 1.56)	14 (10–20)	1.20 (1.01, 1.44)
Incontinent	58 (32%)	5 (2–9)	1.17 (0.91, 1.51)	14 (11–19)	1.18 (1.02, 1.38)

Delirium^c^			*p* = 0.05		*p* = 0.27
No	76 (42%)	6 (2–8)	1.00	14 (9–19)	1.00
Yes	103 (58%)	5 (2–9)	1.27 (1.00, 1.60)	13 (10–18)	1.08 (0.94, 1.24)

Total NPI score (quartiles)			*p* < 0.001		*p* < 0.001
0–14	45 (25%)	3.5 (1–7)	1.00	12 (8–15)	1.00
16–26	45 (25%)	4.5 (2–7)	1.19 (0.87, 1.61)	13 (8–17)	1.07 (0.90, 1.28)
27–41	46 (26%)	6.5 (3–8)	1.53 (1.14, 2.05)	13 (10–18)	1.20 (1.01, 1.44)
42–102	43 (24%)	9 (4–10)	1.91 (1.43, 2.55)	18 (12–22)	1.45 (1.22, 1.72)

NPI frequency					
Delusions			*p* = 0.05		*p* = 0.11
None	83 (46%)	5 (2–8)	1.00	14 (9–18)	1.00
Occ/Often	36 (20%)	4 (1–8)	0.85 (0.63, 1.13)	12 (8–15)	0.92 (0.77, 1.10)
Frequent	29 (16%)	7 (2–10)	1.20 (0.88, 1.65)	12.5 (9–18)	1.00 (0.83, 1.21)
V frequent	31 (17%)	7 (3–10)	1.34 (1.00, 1.81)	14 (12–20)	1.19 (0.99, 1.44)

Hallucinations			*p* = 0.21		*p* = 0.51
None	119 (66%)	5 (2–8)	1.00	13 (9–17)	1.00
Occ/Often	30 (17%)	7 (2–10)	1.19 (0.89, 1.59)	13 (11–20)	1.05 (0.87, 1.26)
Freq/V freq	30 (17%)	6 (3–9)	1.27 (0.94, 1.70)	15.5 (10–19)	1.10 (0.92, 1.32)

Agitation			*p* < 0.001		*p* = 0.02
None	112 (63%)	4 (2–8)	1.00	12 (9–17)	1.00
Occ/Often	36 (20%)	6 (3–8)	1.24 (0.96, 1.60)	13 (11–20)	1.14 (0.97, 1.35)
Freq/V freq	30 (17%)	8 (6–10)	1.73 (1.32, 2.26)	14 (11–21)	1.26 (1.05, 1.50)

Depression			*p* = 0.07		*p* = 0.04
None	60 (34%)	4 (2–7)	1.00	12 (8–16)	1.00
Occ/Often	27 (15%)	5 (3–9)	1.23 (0.86, 1.75)	12.5 (9–19)	1.15 (0.93, 1.40)
Frequent	29 (16%)	7.5 (3–8)	1.23 (0.90, 1.69)	15 (12–20)	1.22 (1.01, 1.48)
V frequent	63 (35%)	7 (3–10)	1.42 (1.10, 1.84)	14 (9–20)	1.23 (1.06, 1.44)

Nervousness			*p* = 0.008		*p* = 0.02
None	64 (36%)	4 (1–7)	1.00	12 (9–16)	1.00
Occ/Often	23 (13%)	4.5 (2–8)	1.22 (0.85, 1.75)	13 (9–16)	1.03 (0.84, 1.26)
Frequent	19 (11%)	7 (5–10)	1.45 (1.00, 2.10)	18 (12–24)	1.37 (1.06, 1.77)
V frequent	72 (40%)	7 (3–10)	1.54 (1.20, 1.97)	14 (9–21)	1.19 (1.02, 1.37)

Elation			*p* = 0.05		*p* = 0.01
None	156 (85%)	5 (2–8)	1.00	13 (9–18)	1.00
Occ/Often/Freq	23 (15%)	8 (5–9)	1.40 (1.01, 1.96)	18 (13–24)	1.26 (1.05, 1.52)

Apathy			*p* = 0.15		*p* = 0.03
None	71 (40%)	4.5 (2–8)	1.00	12 (9–15)	1.00
Occ/Often/Freq	20 (11%)	5 (2–8)	1.11 (0.77, 1.61)	13 (8–21)	1.07 (0.86, 1.33)
V frequent	88 (49%)	6.5 (3–9)	1.26 (1.00, 1.59)	15 (10–20)	1.20 (1.05, 1.38)

Irritability			*p* = 0.006		*p* = 0.02
None	67 (37%)	4 (2–6)	1.00	12 (8–16)	1.00
Occasionally	38 (21%)	5 (2–8)	1.20 (0.89, 1.61)	13.5 (10–20)	1.15 (0.96, 1.36)
Often	21 (12%)	7 (3–8)	1.40 (0.99, 1.97)	13 (11–18)	1.09 (0.87, 1.36)
Frequent	23 (13%)	7 (4–10)	1.50 (1.09, 2.07)	15.5 (10–20)	1.16 (0.94, 1.44)
V frequent	30 (17%)	8 (4–10)	1.72 (1.28, 2.32)	16 (12–21)	1.35 (1.13, 1.62)

Motor behaviour			*p* = 0.005		*p* = 0.007
None	112 (63%)	5 (2–8)	1.00	12 (9–17)	1.00
Occ/Often/Freq	25 (14%)	5.5 (1 –10)	1.04 (0.76, 1.41)	15 (10–20)	1.16 (0.96, 1.40)
V frequent	41 (23%)	8 (4–10)	1.51 (1.18, 1.94)	15.5 (12–22)	1.27 (1.09, 1.49)

Difficulty sleeping			*p* = 0.001		*p* = 0.004
None	91 (51%)	4 (2–7)	1.00	12 (8–16)	1.00
Occ/Often	15 (8%)	8 (2–9)	1.32 (0.92, 1.90)	15 (9–21)	1.17 (0.93, 1.48)
Frequent	30 (17%)	7.5 (5 –10)	1.68 (1.26, 2.25)	15 (11–19)	1.28 (1.07, 1.53)
V frequent	42 (24%)	7 (3–10)	1.52 (1.18, 1.96)	15 (12–21)	1.27 (1.08, 1.49)

Appetite problems			*p* = 0.24		*p* = 0.09
None/Occ	72 (40%)	4 (2–8)	1.00	13 (9–17)	1.00
Frequent	16 (9%)	7 (4–8)	1.18 (0.79, 1.76)	13 (10–20)	1.08 (0.85, 1.38)
V frequent	91 (51%)	6 (3–9)	1.22 (0.97, 1.53)	13 (10–19)	1.16 (1.01, 1.33)

*Note*: Proportional change shows the factor change in scores (or percentage changes if multiplied by 100) in each category relative to the reference category shown by 1. Occ, occasionally; Freq, frequency.

a The overall frequency; numbers in the analysis of CSI and GHQ12 are slightly different because of some carers having missing items on these scales.

b Adjusted for carer sex, age and residence (in relation to patient).

c Delirium Rating Scale score greater than 17.75.

Care home residents were more disabled and behaviourally disturbed than other groups; in particular, apathy was significantly more common among people admitted from care homes (73% compared with 55% in participants living in community). Residents of care homes were also more likely than others to be admitted to trauma wards. Patients with a co-resident carer were somewhat more dependent and had more co-morbidity than those living alone (Table 1).

### Carer characteristics

Fifty-nine per cent of carers were older than 60 years. Half were the son or daughter of the patient, and one quarter were spouses. Non-co-resident carers tended to be sons and daughters, and 48% of this group of carers were in employment. In the other two groups, most carers were retired (Table 2).

**Table 2:** Carer characteristics, strain and psychological distress at recruitment, by carer residence

	Community patients			
			
	Carer lives elsewhere to patient (*n* = 71)	Carer lives with patient (*n* = 58)	Care home patients (*n* = 51)	Total (*n* = 180)
Male^a^	28% (20)	28% (16)	49% (25)	34% (61)
Median age (IQR)^b^	58.5 (50–64)	73 (58.5–78)	64 (59–69)	62 (56–73)
Relationship to patient^b^				
Spouse	0% (0)	66% (38)	12% (6)	24% (44)
Son or daughter	68% (48)	21% (12)	67% (34)	52% (94)
Other relative	24% (17)	14% (8)	18% (9)	19% (34)
Other (non-family)	8% (6)	0% (0)	4% (2)	4% (8)
Employment status^b^				
Employed	48% (34)	9% (5)	18% (9)	27% (48)
Unemployed	13% (9)	14% (8)	14% (7)	13% (24)
Retired	39% (28)	72% (42)	67% (34)	58% (104)
Strain—CSI score^a^				
*n*	66	49	47	162
Median (IQR)	6 (3–9)	7 (5–10)	2 (1–6)	5 (2–8)
Distress—GHQ12^a^				
*n*	67	55	50	172
Median (IQR)	14 (10–19)	14 (11–21)	11.5 (8–15)	13 (9–18.5)
Quality of life—EQ-5D				
*n*	67	56	50	173
Median	0.81	0.73	0.85	0.80
IQR	(0.66–1)	(0.59–0.85)	(0.59–1)	(0.62–1)
Individual EQ-5D items				
Problems walking	27% (19)	35% (20)	24% (12)	28% (51)
Problems washing/dressing	6% (4)	7% (4)	10% (5)	7% (13)
Problems with usual activities^b^	23% (16)	45% (26)	20% (10)	29% (52)
Moderate pain/discomfort	41% (29)	48% (28)	43% (22)	44% (79)
Moderately anxious/depressed^b^	38% (27)	57% (33)	35% (18)	43% (78)

*Note*: Median (IQR) are presented for continuous/ordinal variables and percentage (*n*) for categorical variables.

a Significant difference (*p* < 0.05) between three carer groups.

b Significant difference (*p* < 0.05) between three carer groups and also between the two groups caring for community dwelling participants.

Most carers (72%) said that they were the only person taking care of the patient, although where carers lived elsewhere it was less likely that they were the only person providing care (55%). Co-resident carers spent most time caring (Table 1): 57% indicated that 24-h supervision was required.

Carer strain (CSI) and distress (GHQ12) scores were positively correlated (Spearman's correlation coefficient, 0.63). There were differences in both strain and distress according to carer place of residence: 24% of carers of care home residents having high strain, compared with 45% for non-co-resident and 55% for co-resident carers. Carer quality of life scores (EQ-5D) showed a similar trend although this did not attain statistical significance (*p* = 0.12). Individual EQ-5D items showed that carers often had physical health problems themselves: one third had difficulty with mobility, and one third had difficulty with daily activities; these problems were worse for those carers who resided with the patient (Table 2).

### Association between patient characteristics and carer well-being at baseline

Table 3 shows the associations between carer strain and distress scores at baseline and patient characteristics, which were either statistically significant (using *p*-value < 0.05) or with an adjusted relative effect of 20% or more (proportional change > 1.2 in the table). Patient age, gender, admission ward, co-morbidity score, ADL score prior to the current illness or at admission, and urinary incontinence did not satisfy these criteria for either strain or distress in the unadjusted or adjusted analysis and are therefore not presented in the table. Of the frequency and severity categories used in the assessment of each symptom on the NPI, only frequency is shown in Table 3: the associations with item severities were very similar.

The strongest associations and largest effect sizes for both strain and distress were observed for the total NPI score, with increasing scores as the total NPI score increased. For example, strain scores for carers looking after someone with a total NPI score of 27–42 were 53% greater than for carers of patients with an NPI score between 0 and 14 after adjusting for carer sex, age and residence (adjusted proportional change 1.53, 95% CI 1.14, 2.05). The frequency of symptoms of agitation, anxiety, irritability, motor behaviour problems and difficulty sleeping showed the strongest associations and largest effect sizes when the symptoms assessed on the NPI were considered individually. All of the other individual symptoms, apart from disinhibition, showed trends in the expected direction and had relative effect sizes of greater than 1.2 for either strain or distress. There were also trends for increased strain and/or distress scores for carer of participants with more severe levels of cognitive impairment, symptoms of delirium and faecal incontinence.

### Patient destination at 6 months

At 6 months, 57 (32%) of the patients from the carer–patient dyads originally recruited to the study had died, and a further 23 carers provided no information at follow-up. Small improvements were observed in the strain and psychological distress scores at 6 months compared with admission in the 100 carers who completed follow-up (Table 4).

**Table 4:** Change in carer health at 6 months follow-up by carer residence at admission and patient residence at follow-up (for community residents at admission)

	Community patients			
			
	Carer lives elsewhere to patient (*n* = 71)	Carer lives with patient (*n* = 58)	Care home patients (*n* = 51)	Total (*n* = 180)
Patient mortality	21 (30%)	15 (26%)	21 (41%)	57 (32%)
No follow-up	8 (11%)	10 (17%)	5 (10%)	23 (13%)
Completed follow-up	42 (59%)	33 (57%)	25 (49%)	100 (56%)

Change in CSI				
*n*	37	28	23	88
Median (IQR)	−1 (−3, 0)	−2 (−3.5, 0)	0 (−2, 1)	−1 (−3, 0.5)

Change in GHQ12				
*n*	38	32	24	94
Median (IQR)	−2 (−7, 2)	−3.5 (−6, 0.5)	−1.5 (−5, 1)	−2 (−6, 1)

Change in EQ-5D				
*n*	39	32	24	95
Median (IQR)	0 (−0.15, 0.12)	0.02 (−0.02, 0.17)	0 (0, 0.24)	0 (−0.07, 0.17)

	Community patients (*n* = 129)			
	Remained in community (*n* = 91)		Move to care home (*n* = 38)	
Patient mortality	28 (31%)		8 (21%)	
No follow-up	12 (13%)		6 (16%)	
Completed follow-up	51 (56%)		24 (63%)	

Change in CSI				
*n*	44		21	
Median (IQR)	−1 (−3, 0)		−1 (−4, 0)	

Change in GHQ12				
*n*	48		22	
Median (IQR)	−2 (−5, 2)		−2 (−9, 1)	

Change in EQ-5D				
*n*	50		21	
Median (IQR)	0 (−0.09, 0.16)		0 (−0.12, 0.12)	

*Note*: Change scores calculated as follow-up–admission. Negative values indicate an improvement for CSI and GHQ12 and a deterioration for EQ-5D health status score. In each row, *n* shows the number of carers with complete information on each questionnaire at admission and follow-up.

Thirty-eight patients initially living in the community had moved into a care home at 6 months (29%). Thirty of these patients survived to the end of the study period. There were no differences observed between carers of patients who moved into care homes and carers of patients who remained living in the community who were followed up in terms of change in carer strain, psychological distress or quality of life (Table 4).

## Discussion

There was a high prevalence of strain and psychological distress among the carers studied, and we found differences between groups of carers defined by their living arrangements. People who cared for individuals living in the community were under greater strain and had greater distress at the time of hospital admission than carers of patients who lived in care homes. We found no definite evidence of higher strain in co-resident compared with non-co-resident carers, but co-resident carers often had poor physical health themselves. Patients' behavioural and psychiatric symptoms were strongly associated with both carer strain and distress at baseline. Faecal incontinence was also associated with greater distress and symptoms of delirium with greater strain.

Previous studies on the carers of confused older patients admitted to acute general hospitals are scarce, but our findings are broadly consistent with those of Buck *et al*. (1997). A study from Italy demonstrated greater strain in co-resident compared with non-co-resident carers (Raccichini *et al*., 2009). Our population was older and had more severe cognitive impairment and was therefore not directly comparable, but this may have been due to non-co-resident carers in our study often being sons or daughters, who are more likely to have competing demands from their own children or employment. A study in Spain (Conde-Sala *et al*., 2010) found greater stress among sons and daughters than among spouses, which they attributed to conflicting demands from offspring. Cultural differences in caregiving expectations may also be important (Colombo *et al*., 2011, chapter 3).

A strength of our study was that it was systematic and measured a wide range of patient characteristics and health status problems. We studied patients admitted to an acute general hospital, limiting the general applicability of our findings to all people with dementia. However, this is an important group in policy terms; as they form a large proportion of older people admitted to hospital (Alzheimer's Society, 2009; Sampson *et al*., 2009; Goldberg *et al*., 2012), hospital admissions can cause them disruption and distress, and health services aim to minimise unnecessary hospital admission. Our definition of a carer was broad, but the practicalities of studying patients in acute care settings, which are busy and fast moving, means that recruitment rates were modest. Because of the need for people with mental incapacity to have a consultee, the dyads included in this study may over-represent patients with worse cognition and be biased towards those carers who were able to visit hospital more often. Data on both patients' behavioural and psychiatric symptoms and carer well-being came from the same informant (the carer) at the same interview, so causality cannot be inferred from the observed associations. Our follow-up analysis was limited by lack of statistical power. We did not follow up bereaved carers, who may have had different outcomes.

The strongest associations with poor well-being were disturbed nights and high levels of arousal in the patient (e.g. irritability, agitation). Therefore, to improve carer well-being, interventions to prevent or reduce such behaviours should be investigated and promoted (Robinson *et al*., 2010). Antipsychotic and antidepressant medication are relatively ineffective at achieving these goals (Banerjee, 2009; Banerjee *et al*., 2011). However, in the absence of widespread access to alternatives, these findings may explain why many practitioners feel it necessary to try them. The observed association between Neuropsychiatric Inventory scores and carer strain (CSI) and distress (GHQ12) means that it may be possible to make inferences about carer well-being on the basis of patient assessments. This could be a convenient way of assessing needs for support at a population level, to inform planning and service development. If we know that a patient is displaying disturbed behaviours on the NPI, we should be able to mobilise support for the carer without having to ask the carer questions that may be perceived as unwarranted or intrusive. However, ‘carers’ form a heterogeneous group, making it difficult to generalise about them, and differences in circumstances should be better recognised. By contrast, the EuroQol EQ-5D was not particularly sensitive to carer well-being.

In conclusion, hospital staff should be alert to sources of carer strain and provide practical advice and emotional support for carers. This is important because communicating effectively with carers and finding ways of reducing their stress may improve patient outcomes and reduce care home placement, as 29% of patients initially living in the community had moved to care homes 6 months later. If admissions to long-term care are to be minimised, carer well-being should be a concern of health care providers as well as social services. It may be particularly critical to involve carers at the point when frail older people are admitted to hospital, when carers' knowledge about patients and carer participation in planning can facilitate the treatment phase. Likewise, carers' engagement with acute services can permit their own needs to be appraised and offers an opportunity to improve their capacity to provide ongoing care post-discharge. This ideal scenario calls for an integrated response from primary care, social care, community services and specialist mental health services. The findings from this study highlight how far the general hospital can play a part in such support for carers.

**Key points**

- Carers of people with co-morbid medical and mental health problems living in the community often experience high levels of stress.
- Sleeplessness, agitation and irritability in the person cared for were particularly detrimental to carer well-being.
- Interventions to alleviate behavioural and psychiatric symptoms of dementia could enable carers to cope for longer.
- Carers should be involved in treatment and discharge planning, which should include an assessment of their well-being—the NPI is a reasonable indirect indicator of likely carer strain. The GHQ12 and Carer Strain Index could also serve as direct measures of carer outcome.
